# Prevalence and risk factors of bovine leptospirosis in the Ecuadorian Amazon

**DOI:** 10.14202/vetworld.2024.2612-2618

**Published:** 2024-11-25

**Authors:** Edwin Muyulema, Marcelo Moscoso, Germán Barragán, Roberto Bustillos-Huilca, Jhuliana Luna-Herrera

**Affiliations:** 1 Department of Livestock Sciences, Higher Polytechnic School of Chimborazo (ESPOCH), Riobamba 060106, Ecuador; 2 School of Veterinary Medicine, National University of Loja (UNL), Loja 1101608, Ecuador

**Keywords:** amazon region, bovine, leptospirosis

## Abstract

**Background and Aim:**

Leptospirosis is an infectious zoonotic disease that significantly affects animal health, particularly the reproduction of ruminants. However, some aspects of epidemiology and clinical characteristics have not been clarified. This study aimed to estimate the prevalence and identify risk factors of leptospirosis in female bovines at reproductive age in the Ecuadorian Amazon rainforest.

**Materials and Methods:**

A total of 213 bovines were studied in the Amazon province of Zamora Chinchipe, in which a microscopic agglutination test was used to diagnose a panel of eight serovars of *Leptospira borgpetersenii* (Sejroe) and *Leptospira interrogans*, Australis, Bataviae, Canicola, Tarassovi, Icterohaemorrhagiae, Wolffi, and Hardjo. An epidemiological survey was conducted to identify risk factors by animal and herd and clinical symptoms associated with *Leptospira* spp. infection; and blood samples were collected to determine the differences between seropositive and seronegative animals regarding hematocrit, hemoglobin (Hb), mean corpuscular Hb concentration, total red blood cell count, total platelet count, leukocytes, total proteins, creatinine, and ureic nitrogen.

**Results:**

The prevalence of bovine leptospirosis was 12.21% (26/213), with positive reactions in the Australis, Sejroe, Bataviae, Canicola, and Tarassovi serovars. No variables were considered risk factors, nor clinical signs associated with the infection, nor were there differences in the hematological parameters between the seropositive and seronegative animals.

**Conclusion:**

These findings indicate the persistence of Leptospira on cattle farms in the Ecuadorian Amazon and highlight the interaction between domestic and wild species. It is crucial to implement control measures and improvements in management practices under the One Health approach to reduce accidental infections from contact with wildlife; the awareness of farmers is essential for effective prevention.

## Introduction

Livestock in the Ecuadorian Amazon is constantly growing and face productive problems related to feeding, reproductive management, and animal health [1]. Due to the macroclimate that characterizes this region, some endemic diseases, including leptospirosis, have been reported [2, 3]. Leptospirosis is a zoonotic disease that is globally distributed, and its incidence in humans is highest in tropical regions [4]. According to official figures from the Ecuadorian Ministry of Public Health in 2022 [5], this is the most frequently reported zoonosis in the country, and it is more prevalent in the provinces of the Coast and Amazon regions: Manabí and Zamora Chinchipe, respectively.

Cattle represent a serious health and economic problem due to its consequences on animal reproductive health, given the occurrence of abortions, stillbirths, agalactia, etc. [6]. Previous studies by Barragán [7], Maza [8], and Ruano *et al*. [9] on cattle in Ecuador reported variable prevalences in different serovars of *Leptospira*; however, in the province of Zamora Chinchipe, available information on cattle leptospirosis is scarce. Although the detection of biomarkers is useful in diagnosing, treating, and establishing prognoses in veterinary medicine [10], it is a resource less commonly used by veterinarians for addressing diseases in species of zootechnical interest. In the case of leptospirosis, the clinical laboratory has proven to be diagnostically helpful in domestic species such as dogs as well as in humans with acute cases [11, 12], despite the fact that the type of immune response could depend on the level of exposure, the speed and severity of the animal’s immune response, and the type of serovar infecting the animal [13, 14]. Leptospirosis, which is generally chronic in cattle, typically manifests with abortion [15], which is not easily detected in extensive breeding systems without reproductive records. On the other hand, tissue damage in organs such as the kidneys, liver, and lungs of infected animals has been described postmortem [16] but is rarely manifested clinically. Therefore, hematological and biochemical information can be used to identify damage in organisms that are imperceptible in the silent forms of the disease.

Considering the aforementioned and the coexistence of infectious and non-infectious diseases prevalent in the area that could cause manifestations similar to those of leptospirosis. The study aimed to estimate the prevalence and identify risk factors related to leptospirosis in female cattle of reproductive age in the canton of El Pangui.

## Materials and Methods

### Ethical approval

This study was approved by the Research Committee of the School of Veterinary Medicine (COIF-CMV) of the National University of Loja (UNL-CMV-JAN-2019-0001-O).

### Study period and location

**Figure-1:**
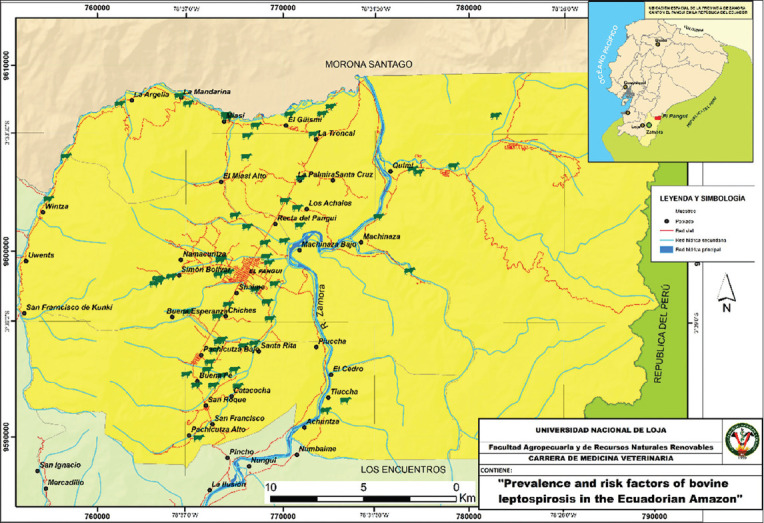
Geographical location of sample farms (green cows) in the canton El Pangui, south-east of the Ecuadorian Amazon region. [Source: The map was generated using QGIS 3.38.3].

The study was conducted from May to July 2019. A descriptive observational and cross-sectional study was carried out in the parishes of Tundayme, El Guismi, El Pangui, and Pachicutza, belonging to the canton El Pangui of the Zamora Chinchipe province, located in the south-east of the Ecuadorian Amazon region (latitude 781816, longitude 9605795 and altitude 820 masl) (Figure-1). The study area has a relative humidity of 90.5%; annual precipitation of 2,285.8 mm, and an average temperature of 21.8°C.

### Sample size

A two-stage sample was designed based on the sampling frame provided by the Agency of Phyto and Zoosanitary Regulation and Control (AGROCALIDAD); the first stage consisted of the aleatory selection of conglomerates represented by farms (primary sampling units); the secondary sampling units were bovine females selected at random proportional to the size of the farm, considering selecting about 25%–75% of the animals of interest on each farm [17]. The number of farms and animals was calculated using the WinEpi 2.0 Software (http://www.winepi.net/menu1.php) using the formula to estimate proportions in finite populations, considering a confidence level of 90%, an expected proportion of infection of 50%, and an absolute error of 10%. In addition, given the research objectives, the calculated sample size was increased by 12%. As a result, 213 blood samples were collected from 66 farms in the four parishes.

This study included female bovines older than 2 years, with no history of leptospirosis vaccination, no antibiotic treatment received 1-month pre-sampling, and no breed condition. Only female bovines were included because the study was designed to investigate reproductive problems, such as abortions and placental retention, and their association with leptospirosis.

### Information record

An epidemiological survey was conducted on each farm. The questionnaire included questions about the exploitation size, replacement source, wetlands presence, type of livestock production, milking system, management system, reproduction system, destination of the milk, water source, presence of other domestic species, rodents, wild animals; and particular data of sampled animals, such as breed and age; on the other hand, it registered information about the presence of clinical signs, such as infertility, abortions, premature births, retained placenta, mastitis, and bloody urine.

### Sample collection and laboratory analysis

Blood samples (~10 mL) were collected in vacutainer with and without anticoagulants by puncturing the caudal vein; the sera obtained were stored at −20°C until shipping. The samples were sent to AGROCALIDAD (Reference Laboratory in Ecuador) for the diagnosis of leptospirosis using the microscopic agglutination test (MAT).

A panel of eight serovars of live antigens of *Leptospira borgpetersenii* Sejroe, *Leptospira interrogans* Australis, Bataviae, Canicola, Tarassovi, Icterohaemorrhagiae, Wolffi, and Hardjo was used. Sera with a single MAT titer of 1/100 were considered seropositive under a dark field microscope (Olympus CX-41, Japan); the positive samples were titrated in double dilutions up to 1/1600; and in case of co-agglutination, the sample was considered positive for the serovar with the highest titer [18]. On the other hand, we determined the values for total proteins, creatinine, blood urea nitrogen, hematocrit (Hto), hemoglobin (Hb), mean corpuscular Hb concentration (MCHC), red blood cells, platelets, and leukocytes (neutrophils and lymphocytes).

### Statistical analysis

The prevalence of bovine leptospirosis was calculated according to the considered categories, i.e., breed, age, parish, farm size, type of production, type of management, other domestic species in contact, animal movement between properties, origin of replacement animals, type of milking, milk destination, breeding system, origin of the breeder, existence of calving areas on the farm, and presence of wetlands on the farm. To establish the relationship between the binary outcome (negativity/positivity to leptospirosis) based on serological analysis results, the chi-square test and/or Fisher’s exact test were performed.

To compare the hematological parameters among the seropositivity, we analyzed the assembled data using the unpaired Student t-test or Wilcoxon test since the data were not normally distributed as determined by the Shapiro–Wilk test. Completed data were exported to a Microsoft Excel 2016 (Microsoft Office, Washington, USA) spreadsheet for cleaning and coding and subsequently transferred to R Software version 4.3.2 (https://www.r-project.org) [19] for subsequent statistical analysis.

## Results

### Prevalence and associated factors

This study estimated the prevalence of 12.21% based on serological analysis using MAT. Titers were recorded between 1/100 and 1/400. These positive reactions were, to a greater extent, related to the serovar Australis and in lesser proportion to the serovars Sejroe, Bataviae, Canicola, and Tarassovi (Table-1).

**Table-1:** Serovars and recorded antibody titers in seropositive animals.

Serovars	Titers of antibodies in MAT						Total	

	1/100		1/200		1/400			
			
	n	%	n	%	n	%	n	%
Australis	7	26.92	2	7.69	2	7.69	11	42.31
Sejroe	4	15.38	1	3.85	1	3.85	6	23.08
Bataviae	1	3.85	3	11.53	1	3.85	5	19.23
Canicola	2	7.69	0	0.00	0	0.00	2	7.69
Tarassovi	2	7.69	0	0.00	0	0.00	2	7.69
Total	16	61.54	6	23.08	4	15.38	26	100

MAT=Microscopic agglutination test

The most seropositive animals belonged to females of Charoláis breed between the ages of 2 and 4 years. Despite these findings, none of these variables were found to be associated with leptospirosis in female bovines in the studied parish (Table-2).

**Table-2:** Prevalence of bovine leptospirosis and associated factors by animal in the El Pangui canton, Ecuadorian Amazon.

Variable	Leptospirosis				p-value

	Negative		Positive		
	
	n	%	n	%	
Breed					0.08
Brown Swiss	40	18.78	7	3.29	
Charoláis	60	28.17	8	3.76	
Girolando	2	0.94	0	0.00	
Holstein Friesian	43	20.19	4	1.88	
Jersey	0	0.00	2	0.94	
Mestiza	42	19.72	5	2.35	
Age					0.28
Group 1 (2–4 years)	78	36.62	14	6.57	
Group 2 (5–8 years)	95	44.60	12	5.63	
Group 3 (9–12 years)	14	6.57	0	0.00	
Total	187	87.79	26	12.21	

The highest prevalence was recorded in animals from El Guismi parish and in livestock farms with the following characteristics: Large livestock farms, rope management, mixed production, self-replacement, manual milking, milk production for family consumption, and livestock farms that move animals between properties of others and their own, with the presence of other wild mammals and presence of wetlands; regarding the reproduction system, the majority of seropositive animals used natural mating, own reproduction, and no farrowing pens (Table-3).

**Table-3:** Prevalence of bovine leptospirosis and associated factors by farm in the El Pangui canton, Ecuadorian Amazon.

Variable	Leptospirosis				p-value

	Negative		Positive		
	
	n	%	n	%	
Parish					0.20
Guismi	12	18.18	5	7.58	
Pachicutza	14	21.21	5	7.58	
Pangui	19	28.79	5	7.58	
Tundayme	2	3.03	4	6.06	
Farm size					0.12
Large	12	18.18	7	10.61	
Medium	18	27.27	10	15.15	
Small	17	25.76	2	3.03	
Production type					0.32
Meat	5	7.58	0	0.00	
Dairy	11	16.67	3	4.55	
Mixed	31	46.97	16	24.24	
The type of management					0.61
Free-range	8	12.12	2	3.03	
Semi-stabled	1	1.52	1	1.52	
Tethering	38	57.58	16	24.24	
Other species in contact					0.15
Equines	5	7.58	0	0.00	
Dogs	10	15.15	9	13.64	
Wild birds	13	19.70	4	6.06	
Wild mammals	19	28.79	6	9.09	
Animal movements between properties					0.66
No	5	7.58	1	1.52	
Yes	42	63.64	18	27.27	
Origin of replacement animals					0.75
Fair	14	21.21	4	6.06	
Others	4	6.06	1	1.52	
Own	29	43.94	14	21.21	
Milking type					0.35
Manual	45	68.18	18	27.27	
Mechanical	0	0.00	1	1.52	
Does not milk	2	3.03	0	0.00	
Milk destination					0.80
Dairy company	11	16.67	5	7.58	
Family supply	31	46.97	14	21.21	
Sold locally	3	4.55	0	0.00	
Calf feed	2	3.03	0	0.00	
Breeding system					0.83
Artificial insemination	5	7.58	2	3.03	
Mixed	9	13.64	2	3.03	
Natural	33	50.00	15	22.73	
Origin of the breeder					0.55
Rented	3	4.55	0	0.00	
Own	44	66.67	19	28.79	
Presence of birthing areas					1
No	46	69.70	19	28.79	
Yes	1	1.52	0	0.00	
Presence of wetlands					0.22
No	1	1.52	1	1.52	
Yes	46	69.70	18	27.27	
Total general	47	71.21	19	28.79	

### Clinical symptoms of *Leptospira* spp. infection

None of the animals with *Leptospira* spp. antibodies presented with the following clinical symptoms in the last year before sampling: bloody urine, preterm birth, or abortion. Most seropositive patients did not present with symptoms such as placental retention or mastitis. However, 17 positive animals had fertility problems. None of these variables were associated with infection (Table-4).

**Table-4:** Clinical symptoms related to the presence of bovine leptospirosis in El Pangui parish, Ecuadorian Amazon.

Clinical symptoms	Leptospirosis				p-value

	Negative		Positive		
	
	n	%	n	%	
Blood in urine					1.00
No	185	86.85	26	12.21	
Yes	2	0.94	0	0.00	
Placental retention					1.00
No	166	77.93	23	10.8	
Yes	21	9.86	3	1.41	
Premature births					0.61
No	177	83.1	26	12.21	
Yes	10	4.69	0	0.00	
Mastitis					1.00
No	169	79.34	24	11.27	
Yes	18	8.45	2	0.94	
Infertility					0.31
No	43	20.19	9	4.23	
Yes	144	67.61	17	7.98	
Abortions					0.61
No	176	82.63	26	12.21	
Yes	11	5.16	0	0. 00	
Total	187	87.79	26	12.21	

### Hematological parameters of *Leptospira* spp. infection

The central tendency measures expressed in Table-5 indicate that urea was the only parameter outside the reference range in the seropositive animals group. None of the considered analytes were registered differences between the studied animal groups.

**Table-5:** Hematological analysis according to the MAT results.

Analyte	Units	Reference range	Leptospirosis				p-value

			Negative		Positive		
	
			Mean	Median	Mean	Median	
Hematocrit	L/L	0.25–0.42	0.37	0.37	0.39	0.39	0.26
Hemoglobin	g/L	80.00–140.00	118.94	118.00	122.62	117.50	0.30
MCHC	g/L	270.00–349.00	320.50	327.80	320.90	329.10	0.72
Leukocytes	×10^9^/L	5.00–10.00	9.65	8.30	9.57	8.90	0.53
Platelets	×10^9^/L	175.00–500.00	508.30	564.00	492.60	530.00	0.88
Granulocytes	×10^9^/L	2.00–6.00	4.20	3.70	4.26	3.80	0.95
Lymphocytes	×10^9^/L	3.00–7.50	4.90	3.90	5.30	3.30	0.48
Total proteins	g/dL	6.20–8.20	7.82	7.70	7.31	7.70	0.84
Urea	mg/dL	7.80–24.60	23.86	23.50	30.38	23.90	0.95
Creatinine	mg/dL	0.60–1.80	0.97	0.93	1.03	0.99	0.12

MAT=Microscopic agglutination test

## Discussion

Epidemiologic information on animal leptospirosis in the Ecuadorian Amazon is scarce, even though this region, mainly the Zamora Chinchipe province, has a significant number of reported cases in humans annually [5].

The results found in this research are lower than those reported in other provinces across the country; for example, in the Loja canton belonging to the province of the same name, adjacent to Zamora Chinchipe, a prevalence of 75% was reported [20]; meanwhile, in the coastal province of Manabí, the seroprevalence at the individual level was 57.38% and at herd level was 97.01% [21]. In addition, the lower prevalence compared with other local and regional studies may be related to chronic infections without detectable antibody titers in MAT. Therefore, it has been suggested that the sensitivity of MAT is dependent on the period between the clinical event and sample collection [22], given that in chronic infections, antibody titers may be below detectable limits. Meanwhile, bacterial isolation or the use of molecular techniques complementary to serology can ensure the identification of animals with leptospirosis [23].

A similar study by Guedes *et al*. [24] conducted in the Amazon region of Brazil (Amazon Delta) recently reported a seroprevalence rate in buffaloes of over 60% using a panel of 34 live antigens. Therefore, the prevalence reported in this investigation could be increased in future research if a larger number of serovars are used, which would increase the sensitivity of the method. However, beyond the MAT sensitivity, it is also likely that the results are due to the cattle management system, which in this study area is mostly free range or halter rope used for handling the animals (“sogueo”), so the possibility of transmission of infectious agents decreases considerably.

As previously reported in Ecuador [20], the serovar Australis was determined with considerable frequency; in this regard, researchers around the world have indicated that opossums, rodents, sheep, cats, etc., serve as hosts for this serovar [25, 26]. Although rodents are known reservoirs of the serovar Bataviae, incidental infections by serovars such as Tarassovi have also been detected, whose maintenance hosts are pigs [6, 27, 28]. Regarding the serovars Sejroe and Canicola, some wild and domestic carnivorous mammals have been reported as maintenance hosts [6, 29, 30].

Given the diversity of serovars reported in this study, as well as the probable maintenance hosts, the importance of the interaction between domestic and wild animals in the epidemiology of this disease is evident. However, in Ecuador, it is not yet known with certainty which animal species could act as reservoirs of the serovars that cause disease in domestic animals; consequently, this should be taken into account in future research.

Despite the results obtained regarding the factors associated with this study, it is clear that the biosafety measures used on farms are decisive when it comes to preventing diseases of infectious origin; thus, some practices could become risk factors for the disease in the study area, such as the transfer of animals between farms or the replacement of animals from an unknown origin.

Although vaccination is considered a key pillar for controlling the disease, the prevention of renal colonization through immunization remains controversial. Thus, according to Wilson-Welder *et al*. [31], *Leptospira* spp. was not isolated in experimentally infected cows after vaccination, which is consistent with the findings of Ruano *et al*. [9]. However, in other post-vaccination studies by Aymée *et al*. [32] and Sonada *et al*. [33], the presence of the pathogen was identified, leading to the suggestion that the use of bacterins may not prevent renal and genital colonization.

Although the density of livestock is indeed controlled by traditional management systems in the Amazon, it must also be considered that direct or indirect contact with wild species could facilitate the transmission of the disease in areas that, due to their geographical and environmental characteristics, favor the persistence of the bacteria in the medium.

Hematology involves the use of multiple tools to determine an appropriate diagnosis in ruminants; regarding leptospirosis, some alterations have been suggested, such as hemolysis or thrombocytopenia during the acute stage [34]; however, studies in cattle have not been conclusive regarding hematological alterations with diagnostic value in this species, particularly in the chronic phase; despite no statistical differences being found in the parameters analyzed between seropositive and seronegative animals, another study by Ijaz *et al*. [35] has mentioned significant differences in parameters such as Hb and creatinine.

A similar study by Vihol *et al*. [36] carried out in goats reported a highly significant decrease in Hto, Hb, and MCHCs, as well as a decrease in total protein measurement in seropositive animals versus seronegative animals; although, as indicated in this research, no differences were found in the parameters of the renal profile, which is probably because renal function continues until the tissue enters a point of no return. In horses, significant differences have been reported between seropositive and seronegative animals in parameters such as Hb, Hto, MCHC, total erythrocyte count, platelet count, and leukocyte count [37].

All of these investigations agreed that the alterations in the eritrogram were due to the virulence mechanisms of the bacteria, particularly hemolysis generated by certain types of serovars and vasculitis, which is the initial damage during pathogenesis. Despite the results presented in this study, the use of hematology as a resource in the diagnosis of infectious diseases to which ruminants are susceptible should not be left aside.

The results on bovine leptospirosis presented in this study are relevant to the field of public health, as leptospirosis is considered an endemic disease in Ecuador, with epidemic outbreaks particularly associated with prolonged periods of rain and flooding in the Coastal and Amazon regions [38]. According to a study by Calero and Monti [39], the leptospirosis surveillance system in Ecuador is sufficiently sensitive for decision-making and monitoring in high-risk areas, such as coastal provinces; however, it shows weaknesses in other geographic regions of the country, suggesting that human leptospirosis remains an underdiagnosed and underreported disease. In light of this scenario, the need for interdisciplinary collaboration under a One Health perspective to strengthen health surveillance systems for zoonoses becomes evident again [40].

## Conclusion

The seroprevalence of bovine leptospirosis in El Pangui parish was 12.21%, with seropositive animals having been identified for the serovars Australis, Sejroe, Bataviae, Canicola, and Tarassovi, with titers between 1/100 and 1/400, which evidence the permanence of *Leptospira* in cattle farms in the Ecuadorian Amazon region, as well as the interaction between domestic and wild species responsible for incidental infections. No factors, clinical signs, or hematological parameters were associated with *Leptospira* spp. infection. The results of this study highlight the need to improve cattle management practices in the Amazon region. Farmers should be educated about the risks associated with leptospirosis and the preventive measures they can take to protect their animals and themselves. To monitor the disease, local authorities and veterinary services should also collaborate from a One Health approach, this information should be considered to rethink the surveillance actions maintained by the Ministry of Public Health over the population, including active case-finding, especially among people closely involved in handling infected animals, as they are at constant risk of bacterial exposure.

## Authors’ Contributions

JLH, MM, and RBH: Conceptualized the study. JLH, GB, and EM: Conducted the experiments, laboratory work, and data collection. EM: Sample collection. EM and RBH: Data analysis. JLH, MM, GB, and RBH: Study supervision and manuscript revision. EM and JLH: Drafted the manuscript. All authors have read and approved the final manuscript.
